# A glycometabolic gene signature associating with immune infiltration and chemosensitivity and predicting the prognosis of patients with osteosarcoma

**DOI:** 10.3389/fmed.2023.1115759

**Published:** 2023-05-24

**Authors:** Fengyan Wang, Kun Yang, Runsang Pan, Yang Xiang, Zhilin Xiong, Pinhao Li, Ke Li, Hong Sun

**Affiliations:** ^1^Department of Orthopaedics, The Affiliated Hospital of Guizhou Medical University, Guiyang, China; ^2^School of Basic Medicine, Guizhou Medical University, Guiyang, China; ^3^School of Clinical Medicine, Guizhou Medical University, Guiyang, China; ^4^Department of Pathology, The Affiliated Hospital of Guizhou Medical University, Guiyang, China; ^5^Department of Respiratory and Critical Care Medicine, Guizhou Provincial People’s Hospital, Guiyang, China

**Keywords:** osteosarcoma, glycometabolism, prognosis, immune infiltration, chemotherapy

## Abstract

**Background:**

Accumulating evidence has suggested that glycometabolism plays an important role in the pathogenesis of tumorigenesis. However, few studies have investigated the prognostic values of glycometabolic genes in patients with osteosarcoma (OS). This study aimed to recognize and establish a glycometabolic gene signature to forecast the prognosis, and provide therapeutic options for patients with OS.

**Methods:**

Univariate and multivariate Cox regression, LASSO Cox regression, overall survival analysis, receiver operating characteristic curve, and nomogram were adopted to develop the glycometabolic gene signature, and further evaluate the prognostic values of this signature. Functional analyses including Gene Ontology (GO), kyoto encyclopedia of genes and genomes analyses (KEGG), gene set enrichment analysis, single-sample gene set enrichment analysis (ssGSEA), and competing endogenous RNA (ceRNA) network, were used to explore the molecular mechanisms of OS and the correlation between immune infiltration and gene signature. Moreover, these prognostic genes were further validated by immunohistochemical staining.

**Results:**

A total of four genes including *PRKACB*, *SEPHS2*, *GPX7*, and *PFKFB3* were identified for constructing a glycometabolic gene signature which had a favorable performance in predicting the prognosis of patients with OS. Univariate and multivariate Cox regression analyses revealed that the risk score was an independent prognostic factor. Functional analyses indicated that multiple immune associated biological processes and pathways were enriched in the low-risk group, while 26 immunocytes were down-regulated in the high-risk group. The patients in high-risk group showed elevated sensitivity to doxorubicin. Furthermore, these prognostic genes could directly or indirectly interact with other 50 genes. A ceRNA regulatory network based on these prognostic genes was also constructed. The results of immunohistochemical staining showed that *SEPHS2*, *GPX7*, and *PFKFB3* were differentially expressed between OS tissues and adjacent normal tissues.

**Conclusion:**

The preset study constructed and validated a novel glycometabolic gene signature which could predict the prognosis of patients with OS, identify the degree of immune infiltration in tumor microenvironment, and provide guidance for the selection of chemotherapeutic drugs. These findings may shed new light on the investigation of molecular mechanisms and comprehensive treatments for OS.

## 1. Introduction

Osteosarcoma (OS) is derived from primitive osteogenic mesenchymal cells, which is recognized as the most common type of malignant bone tumor in childhood and adolescence (1). OS prefers to occur in the metaphysis of long bones, especially around the knees, and is characterized by high rates of metastasis and progression (2). With the improvement of neoadjuvant therapy and surgical resection, more than two-thirds of patients with localized lesions are likely to achieve long-term survival (3, 4). However, approximately 30% of the patients with non-metastasis at diagnosis suffer from lung metastasis after comprehensive treatments (5). What’s worse, the 5-year survival rate of the patients with distant metastasis at diagnosis is still unfavorable, and patients with metastatic OS or chemoresistance are lacking of the effective therapeutic interventions (6). It is believed that early detection and intervention is of great importance to improve the overall survival of patients with OS. Thus, novel prognostic biomarkers are urgently needed for the diagnosis and treatment of OS.

Glycometabolism is a universal pathway involved in cell growth and survival. Normal and non-proliferating cells obtain energy though oxidative phosphorylation under aerobic conditions (7). Whereases cancer cells mainly depend on aerobic glycolysis for adenosine triphosphate (ATP) production for the requirement of rapid proliferation and invasion, and display enhanced glucose uptake for the compensation against the low energy yield of aerobic glycolysis (8, 9). Increasing studies have shown that elevated glucose uptake and aerobic glycolysis in cancer cells is associated with distant metastasis and unfavorable prognosis (10). Recent studies also have found that dysregulation of glycometabolism is involved in tumorigenesis and treatment of OS (11–13). Increased aerobic glycolysis facilitates cell growth, metastasis, and chemoresistance in OS (14, 15). Targeting aerobic glycolysis may be an attractive therapeutic option for the treatment of OS (16, 17). However, the mechanisms of glycometabolic genes in OS remain largely unknown. Recently, several signatures based on glycometabolic genes and lncRNAs have been established in multiple tumors, and these signatures can contribute to elucidate the association between glycometabolism and prognosis (18–21). Nevertheless, the study on glycometabolic gene signature in OS still remains limited.

In this study, we obtained the expression profiles and corresponding clinical information of OS patients from the therapeutically applicable research to generate effective treatments (TARGET) database and the gene expression omnibus (GEO) database and then constructed a novel glycometabolic gene signature to predict the clinical outcomes of patients with OS. Meanwhile, the immune status and chemotherapy drug sensitivity between high- and low-risk groups were also evaluated. Finally, the potential mechanisms of these prognostic genes in tumorigenesis of OS and their up-regulatory network were further investigated. These findings may provide novel prognostic biomarkers and molecular mechanisms for the diagnosis and treatment of OS.

## 2. Materials and methods

### 2.1. Selection of datasets and data acquisition

**FIGURE 1:**
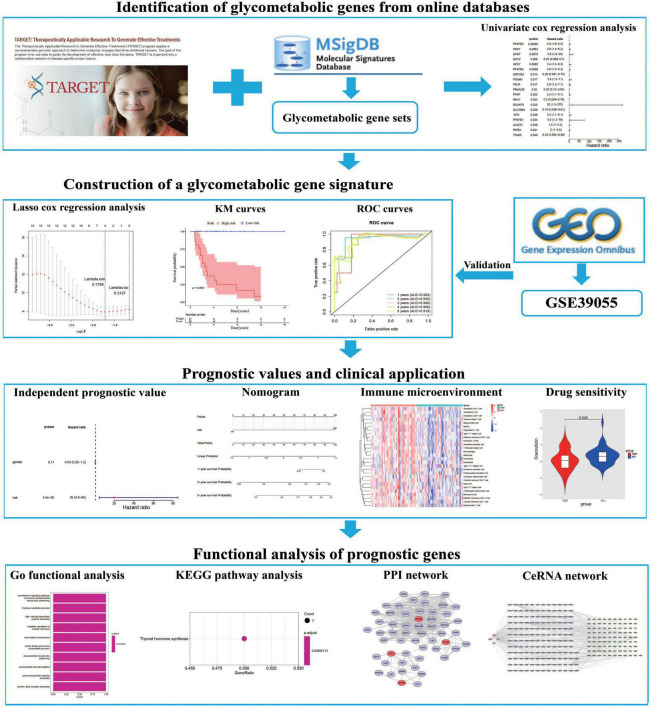
The flowchart of this study.

The expression profile and corresponding clinical data of OS patients were downloaded from the TARGET database (22).^1^ The dataset includes 85 OS patients with survival information, which were then randomly separated into training (*n* = 43) and testing (*n* = 42) cohorts at cut-off 5:5. Moreover, the GSE39055 dataset which contains 36 OS patients with survival information were downloaded from the GEO database^2^ as a validation cohort. Furthermore, we extracted 8 glycometabolic gene sets from the molecular signatures database (MSigDB) (23).^3^ As shown in Supplementary Table 1, the entire glycometabolic gene set contained 291 genes after removing overlapping genes. There were 282 genes left after removing genes whose expression is 0 in 50% of the samples. The workflow chart of this study is shown in Figure 1.

### 2.2. Construction and validation of a glycometabolic gene signature

Univariate Cox analysis was performed to assess the prognostic value of glycometabolic genes. Genes with a *P*-value < 0.05 were considered as potential prognostic genes. Next, the LASSO regression algorithm was used to construct an optimal glycometabolic gene signature. The risk score for each patient was calculated as follows: risk score = $\sum_{i=1}^{n}\beta i\times Expgene(i)$, where *n* is the number of genes in this prognosis model, *β* represents the regression coefficient, and *Expgene* is the expression level of each gene. We divided the patients from the training cohort (*n* = 43) into high- and low-risk groups based on the median value of the risk score, that individuals in the high-risk groups suffered from a lower survival probability and higher risk of death compared to that in low-risk group. Differences of survival probability between these two risk groups were assessed using Kaplan–Meier (KM) curves. Receiver operating characteristic (ROC) curves were used to evaluate the prognostic capacity of the gene signature. The gene signature was further tested and validated in the testing (*n* = 42) and validation cohort (*n* = 36), respectively.

### 2.3. Independent prognostic analysis and construction of a nomogram

The univariate and multivariate Cox regression analyses were used to identify independent prognostic factors in the TARGET dataset. The correlation between risk score and metastasis was evaluated using chi-squared test. The results of multivariate regression analysis were used to establish a nomogram to predict the 1-, 3-, and 5-year overall survival. Moreover, the discrimination and accuracy of the nomogram were assessed by the concordance index (C-index), calibration curves, and decision curves, respectively.

### 2.4. Functional enrichment analysis

The “limma” package was used to analyze differentially expressed genes (DEGs) between the high- and low-risk groups in the TARGET dataset. Gene set enrichment analysis (GSEA) was performed to determine the biological functions of DEGs. Gene Ontology (GO) and kyoto encyclopedia of genes and genomes (KEGG) pathway enrichment analyses were performed using the ClusterProfiler package (24, 25). Moreover, we also explored the single-sample GSEA (ssGSEA) scores between the high- and low-risk groups based on gene expression profiles involved in the top 10 GO and KEGG terms.

### 2.5. Analysis of immune infiltration

To analyze the differences in the proportion of 28 immunocytes between the high- and low-risk groups, ssGSEA was performed using TARGET dataset (26). We also investigated the correlation between the proportion of the 28 immunocytes and prognostic genes. Tumor immune infiltration scores including immune score, stromal score, ESTIMATE score, and tumorpurity were assessed using the “estimate” and “limma” packages.

### 2.6. Drug sensitivity analysis

The R package “pRRophetic” was used to calculate the half-maximal inhibitory concentration (IC50) of chemotherapy drugs, and the Wilcoxon signed-rank test was employed to compare the differences of IC50 between the high- and low-risk groups.

### 2.7. Protein-protein interaction (PPI) network

To explore the interactions among these prognostic genes, a PPI network was constructed using the online tool search tool for the retrieval of interacting genes (STRING) database (27). Furthermore, a plug-in of Cytoscape software (version 1.6.20), molecular complex detection (MCODE), was used to screen the significant modules in the PPI network (28).

### 2.8. Construction of a ceRNA network

MiRNAs that can regulate prognostic genes were predicted based on miRanda software. Then, lncRNAs that can regulate the predicted miRNAs were predicted through miRanda software. To improve the accuracy of the competing endogenous RNA (ceRNA) network, we further screened the results using the following criteria: combined score > 200 was set as the screening criteria of lncRNA-miRNA interactions, combined score > 200 and minimum free energy (MFE) score < −200 were set as the screening criteria of miRNA-gene interactions. Finally, a ceRNA network was constructed using Cytoscape.

### 2.9. OS samples and immunohistochemical staining

This study was approved by the Ethics Committee of Affiliated Hospital of Guizhou Medical University (No. GZYD003-201753035). The tumor samples and corresponding adjacent normal tissues were obtained from the patients who were diagnosed with OS. All the clinical information of patients were shown in Supplementary Table 2. Human samples were fixed with 4% paraformaldehyde, embedded in paraffin, and sliced into 5-μm sections. Sections were deparaffinized in xylene, hydrated with a graded ethanol series at room temperature. Next, sections were treated with 3% H_2_O_2_ to block endogenous peroxidase activity, and then blocked with 5% bull serum albumin (BSA) for 30 min at room temperature. The specimens were incubated with the appropriate primary antibodies at 4 °C overnight and incubated with goat anti-rabbit secondary antibodies (1:200, Proteintech, Wuhan, China, SA00001-2) at 37 °C for 2 h at room temperature. Primary antibodies used in this experiment included antibodies against *PRKACB* (1:200, Proteintech, 12232-1-AP), *SEPHS2* (1:200, Proteintech, 14109-1-AP), *GPX7* (1:200, Proteintech, 13501-1-AP), and *PFKFB3* (1:200, Proteintech, 13763-1-AP). The DAB substrate system (Solarbio, China) was used for color development, and hematoxylin staining was used to reveal the cell nuclei. Images were obtained under a light microscope.

### 2.10. Statistical analysis

All the above analyses were completed by the R software (Version 4.0.5). A time-dependent ROC analysis was performed by the “pROC” package (29). Chi-squared test was used to explore the correlation between risk score and metastasis of OS. And a nomogram was constructed by the “rms” packages. Results with a *P*-value < 0.05 were considered as statistical significance.

## 3. Results

### 3.1. Construction of a glycometabolic gene signature in OS

**FIGURE 2:**
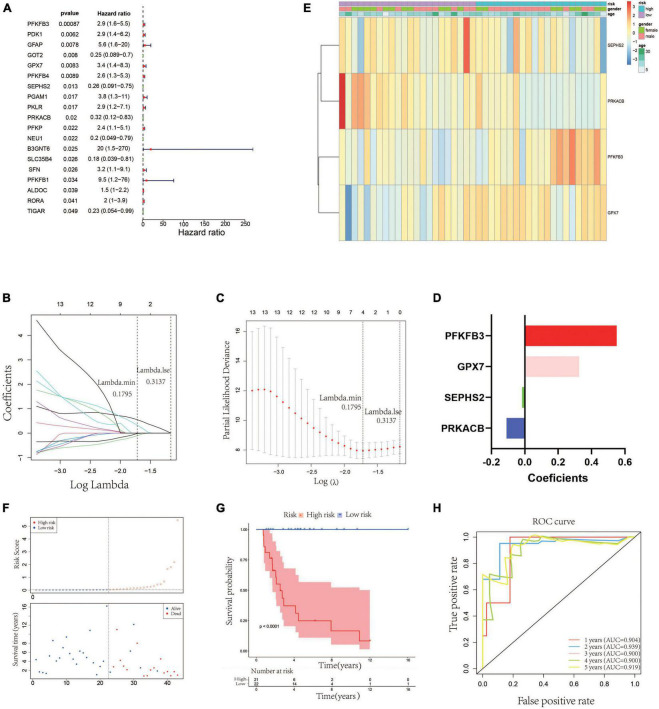
Construction of a glycometabolic gene signature in the training cohort. **(A)** Univariate Cox regression analysis of glycometabolic genes. **(B)** LASSO Cox regression analysis of glycometabolic genes. **(C)** Selection of the optimal penalty parameter for LASSO Cox regression. **(D)** Coefficients of the four glycometabolic genes though LASSO Cox regression. **(E)** Heat map visualizing the expression of the four glycometabolic genes between risk groups. **(F)** The scatter plot of patients’ risk score and survival time. **(G)** Kaplan–Meier curves of patients in low- and high-risk groups. **(H)** Time-dependent ROC curves of 1-, 2-, 3-, 4-, and 5-years survival.

In order to establish a glycometabolic gene signature, we identified 19 glycometabolic genes which were significantly associated with the overall survival of the patients with OS in the training cohort using univariate Cox regression analysis (Figure 2A). Subsequently, LASSO Cox regression analysis was performed to screen candidate genes for constructing the gene signature. The results indicated that 4 glycometabolic genes, including protein kinase CAMP-activated catalytic subunit beta (*PRKACB*), selenophosphate synthetase 2 (*SEPHS2*), glutathione peroxidase 7 (*GPX7*), and 6-phosphofructo-2-kinase/fructose-2,6-biphosphatase 3 (*PFKFB3*), were identified according to the optimum penalty parameter (λ) value (Figures 2B, C). The coefficient of each candidate gene is shown in Figure 2D. Combining the gene expression with corresponding regression coefficient, a risk score model was then established using a formula as follows: risk score = expression level of *PRKACB* × (−0.10894429) + expression level of *SEPHS2* × (−0.01563243) + expression level of *GPX7* × 0.32559346 + expression level of *PFKFB3* × 0.55009921. According to the median value of the risk scores, the patients in the training cohort were divided into high- (*n* = 21) and low-risk group (*n* = 22). The expression of *GPX7* and *PFKFB3* had a positive correlation with risk scores, while the expression of *SEPHS2* and *PRKACB* was negatively associated with risk scores (Figure 2E). However, the expression of these four genes was not associated with age and gender. As shown in Figure 2F, the patients with high-risk scores seemed to have high mortality rates and shorter survival time than those with low-risk scores. Similarly, the KM curves showed that patients in the low-risk group had a better clinical prognosis than those in the high-risk group (Figure 2G). Furthermore, the respective area under the ROC curve (AUC) of 1-, 2-, 3-, 4- and 5-year survival was 0.904, 0.939, 0.900, 0.900, and 0.919, suggesting that this glycometabolic gene signature showed a favorable prognostic value of overall survival (Figure 2H).

### 3.2. Validation of the glycometabolic gene signature

**FIGURE 3:**
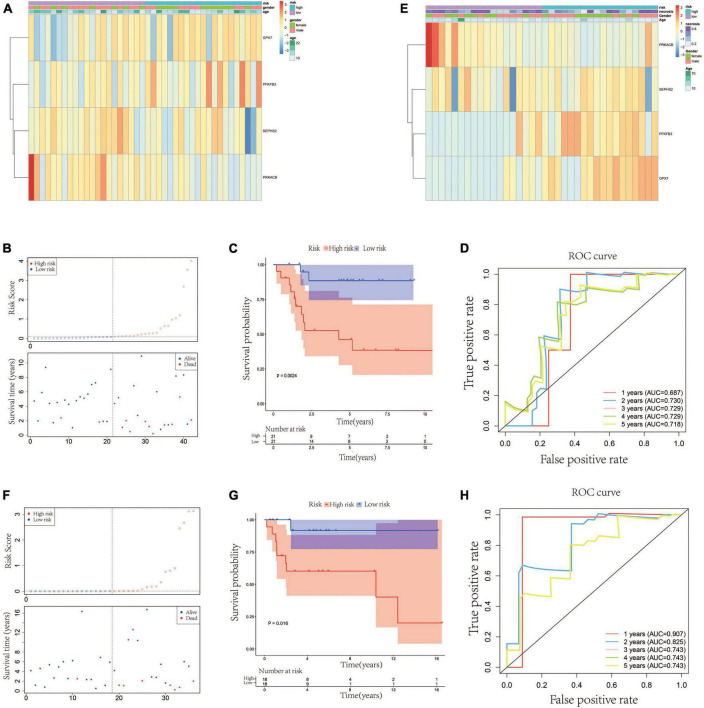
Validation of the glycometabolic gene signature in testing and validation cohorts. **(A)** Heat map visualizing the expression of the four glycometabolic genes between risk groups in testing cohort. **(B)** The scatter plot of patients’ risk score and survival time in testing cohort. **(C)** Kaplan–Meier curves of patients in testing cohort. **(D)** Time-dependent ROC curves of 1-, 2-, 3-, 4-, and 5-years survival in testing cohort. **(E)** Heat map visualizing the expression of the four glycometabolic genes between risk groups in validation cohort. **(F)** The scatter plot of patients’ risk score and survival time in validation cohort. **(G)** Kaplan–Meier curves of patients in validation cohort. **(H)** Time-dependent ROC curves of 1-, 2-, 3-, 4-, and 5-years survival in validation cohort.

According to the median value of the risk scores, the 42 patients in the testing cohort were equally separated into high- and low-risk groups. The heatmap visualized the expression of *GPX7*, *PFKFB3*, *SEPHS2*, and *PRKACB* in high- and low-risk groups. The results showed that the expression patterns of these 4 prognostic genes in testing cohort were similar to that in training cohort (Figure 3A). The risk scatter plots and KM survival analysis also indicated that the patients with high-risk scores had worse prognoses than those with low-risk scores (Figures 3B, C). The AUC was 0.687 at 1-year, 0.730 at 2-year, 0.729 at 3-year, 0.729 at 4-year, and 0.718 at 5-year (Figure 3D). In order to verify the robustness of this prognostic signature, GSE39055 which contains 36 OS patients was used as an external validation cohort. The risk score of each patient in validation cohort was calculated using the same formula determined in training cohort. The 36 patients in the validation cohort were equally separated into high- and low-risk groups. The expression patterns of these 4 prognostic genes were depicted on the heatmap which also showed similarity to that in training cohort (Figure 3E). As shown in Figures 3F, G, poor prognosis was found in patients with high-risk scores, and the percentages of dead patients were 50% (9/18) and 5.6% (1/18) in the high- and low-risk groups, respectively. The respective AUC was 0.907, 0.825, 0.743, 0.743 and 0.743 for 1, 2-, 3-, 4-, and 5-years survival (Figure 3H). All these results indicated that this glycometabolic gene signature had a robust performance in predicting the prognosis of patients with OS.

### 3.3. Independent prognostic value of the glycometabolic gene signature and construction of a nomogram

**FIGURE 4:**
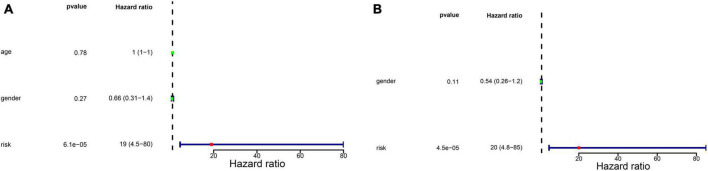
Independent prognostic value of glycometabolic gene signature. **(A)** Univariate Cox regression analysis and **(B)** multivariate Cox regression analysis of the correlations between prognosis and age, gender, and risk score.

We firstly analyzed the correlation between risk score and metastasis. The results revealed that the proportion of individuals with metastasis in the high-risk group was more than that in low-risk group (Supplementary Figure 1). To further assess the independent prognostic value of the glycometabolic gene signature, we conducted univariate and multivariate Cox regression analyses based on the risk score and clinical characteristics in TARGET dataset. Univariate Cox regression analysis identified that the risk score could serve as a survival related variable (Figure 4A). Subsequently, multivariate Cox regression analysis was used to determine the independent prognostic value of the risk score, and the results showed that the risk score was an independent prognostic factor (Figure 4B). Thus, these results demonstrated that the risk score was significantly associated with metastasis, and could serve as an independent prognostic factor for patients with OS.

**FIGURE 5:**
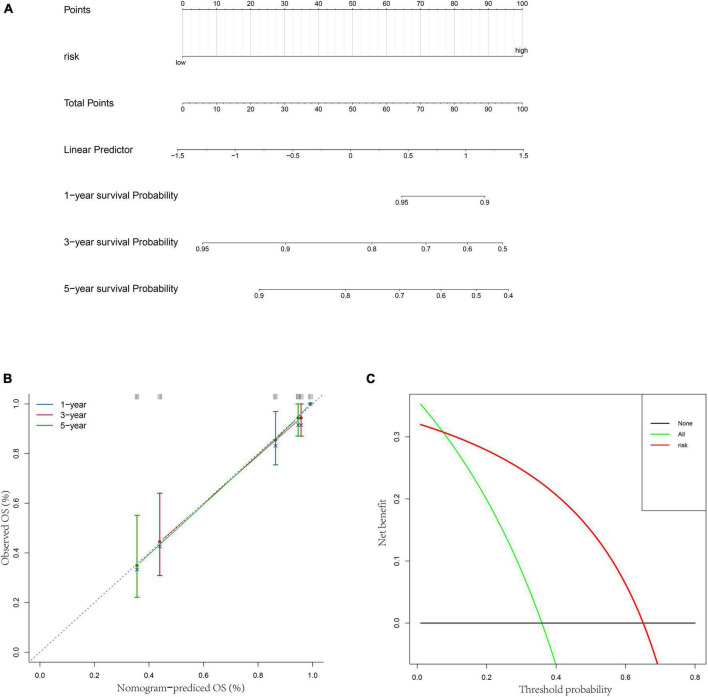
Construction of a nomogram based on risk score. **(A)** A nomogram for predicting 1/3/5-year survival rates of OS patients. **(B)** 1/3/5-year calibration curves of the nomogram. **(C)** Decision curve of the risk score.

Besides, to better provide an applicable quantitative tool for clinic practice, we constructed a new nomogram based on the independent prognostic factor to predict the prognosis of patients with OS in TARGET dataset at different years after diagnosis. Then, total points of each patient could be calculated according to the survival rate at 1, 3-, and 5-years (Figure 5A). The results showed that the overall survival of patients at 1, 3-, and 5-years reduced along with the increase of total scores. The C-index was 0.76 and the calibration curve was similar to the ideal curve (Figure 5B). Furthermore, decision curves were drawn to assess the clinical utility of the risk score model. The results showed that the risk score model could yield more net benefit for predicting the 5-year survival rates than both treat-all and treat-none strategies (Figure 5C). All these findings indicated that the nomogram showed favorable predictive ability and application value.

### 3.4. The expression of prognostic genes and their prognostic values

**FIGURE 6:**
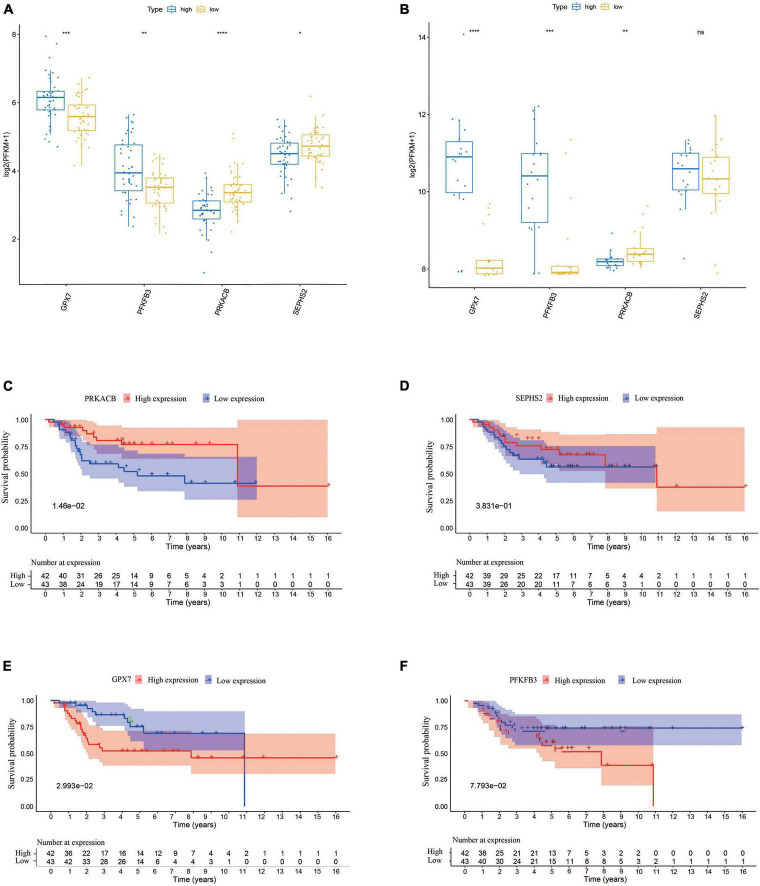
The expression of the prognostic genes and their prognostic values. **(A)** The expression of the prognostic genes in TARGET dataset. **(B)** The expression of the prognostic genes in validation cohort. **(C–F)** The Kaplan–Meier curves for overall survival of osteosarcoma patients in the high and low *PRKACB* **(C)**, *SEPHS2* **(D)**, *GPX7* **(E)**, and *PFKFB3* **(F)** expression groups from the TARGET dataset.

In order to understand the prognostic values of these four glycometabolic genes, we firstly investigated the expression of each gene between high- and low-risk groups in TARGET dataset. The expression of *PFKFB3* and *GPX7* was upregulated while the expression of *PRKACB* and *SEPHS2* was downregulated in high-risk group when compared to that in low-risk group (Figure 6A). The expression trends of these prognostic genes in validation cohort were similar to that in TARGET dataset except for *SEPHS2* (Figure 6B). Then the prognostic values of glycometabolic genes were evaluated in TARGET dataset. As a result, the elevated expression of *PRKACB* was associated with favorable clinical outcomes (Figure 6C), whereas the patients with high expression of *GPX7* had poorer prognosis (Figure 6E). Nevertheless, there was no distinct difference between the expression level and overall survival in terms of *SEPHS2* and *PFKFB3* (Figures 6D, F). These findings showed that glycometabolic genes including *PRKACB* and *GPX7* were significantly correlated with the prognosis of patients with OS.

### 3.5. Functional enrichment analyses of DEGs based on risk score

**FIGURE 7:**
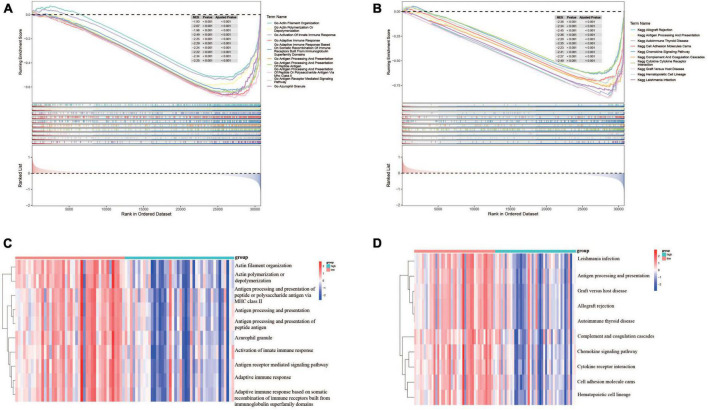
Functional enrichment analyses of DEGs between risk groups. **(A)** The top 10 GO terms enriched in the low-risk group using GSEA analysis. **(B)** The top 10 KEGG terms enriched in the low-risk group using GSEA analysis. **(C)** Heatmap of the top 10 GO terms between risk groups using ssGSEA analysis. **(D)** Heatmap of the top 10 KEGG terms between risk groups using ssGSEA analysis.

In order to understand the potential biological functions, the DEGs based on risk score were explored in the TARGET dataset. As shown in Supplementary Table 3, a total of 940 DEGs including 336 upregulated genes and 604 downregulated genes were identified between risk groups. Subsequently, we conducted GSEA to explore the discrepancies of biological processes and pathways between two risk groups. The results of GO enrichment analysis indicated that DEGs were enriched in multiple biological processes including activation of innate immune response, adaptive immune response, antigen processing and presentation, and antigen receptor-mediated signaling pathway (Supplementary Table 4). The top 10 enriched biological processes are shown in Figure 7A. Results from KEGG analysis showed that the DEGs were mainly involved in allograft rejection, antigen processing and presentation, autoimmune thyroid disease, chemokine signaling pathway, complement and coagulation cascades, and cytokine-cytokine receptor interaction (Supplementary Table 5). The top 10 enriched pathways are shown in Figure 7B. The above results suggested that these DEGs were involved in various immune related processes and pathways, indicating widespread correlation between glycometabolism and immune status. In addition, we further calculated the scores of each biological process and pathway in each patient using ssGSEA. The heatmaps depicted the top 10 biological processes and pathways with significant differences of scores between the two risk groups, respectively (Figures 7C, D). The results revealed that several immune related processes and pathways were downregulated in high-risk group.

### 3.6. Evaluation of immune microenvironment characteristics between risk groups

**FIGURE 8:**
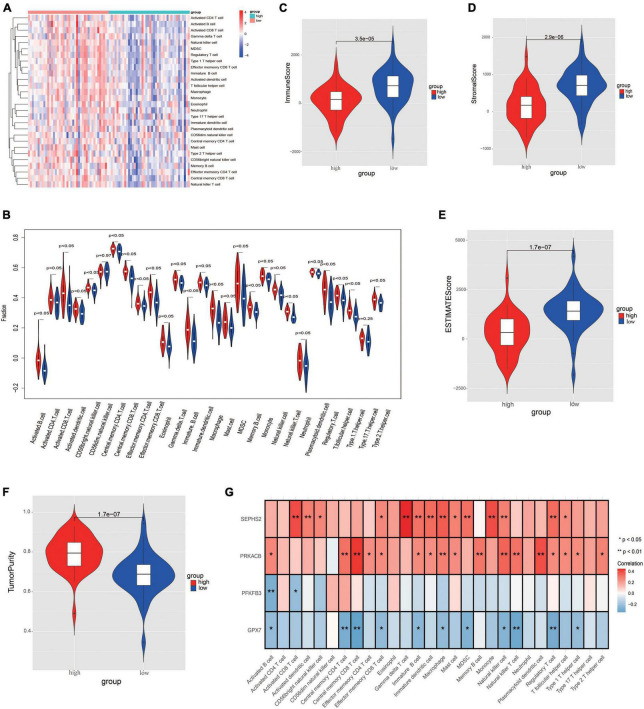
Evaluation the differences of immune microenvironment between risk groups. **(A)** Heatmap of immune cell populations between risk groups. **(B)** Proportion of immune cell subpopulations between risk groups. **(C–F)** Immune scores **(C)**, stromal score **(D)**, ESTIMATE score **(E)**, and tumorpurity score **(F)** between risk groups. **(G)** Heatmap visualizing the correlation between the four prognostic genes and immune cell subpopulations.

In order to further evaluate the correlation between immune and the glycometabolic gene signature, we next carried out ssGSEA to analyze the immune infiltration in TARGET dataset. As shown in Figure 8A, the heatmap described the abundance of 28 immunocytes between the two risk groups. A total of 26 immunocytes were significantly decreased in the patients with high-risk scores in comparison to the patients with low-risk scores (Figure 8B). Higher level of Type 17 T helper cell was found in low-risk group while there was no significant difference between the two risk groups. Moreover, the immune infiltration scores and tumorpurity were also assessed between two risk groups. As shown in Figures 8C–F, the immune score, stromal score, and ESTIMATE score were higher while the tumorpurity was lower in low-risk group when compared to those in high-risk group. Besides, the correlation between these 4 prognostic genes and the immunocytes was further analyzed. The results showed that the *SEPHS2* was positively correlated with 14 immune cells, *PRKACB* was positively correlated with 17 immune cells, *GPX7* was negatively correlated with 11 immune cells, and *PFKFB3* was negatively correlated with 2 immune cells, respectively (Figure 8G).

### 3.7. Analysis of chemotherapy drugs sensitivity between risk groups

**FIGURE 9:**
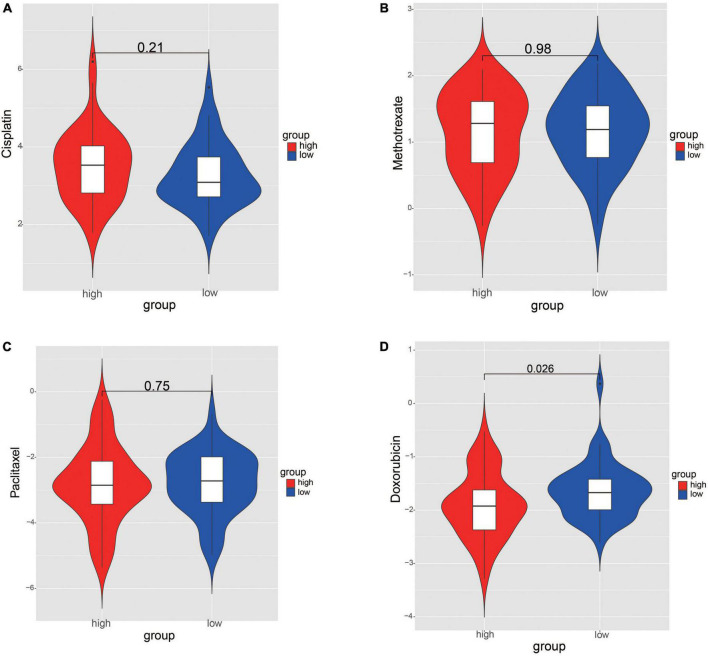
Comparation the differences of chemotherapy drug sensitivity between risk groups. **(A)** Cisplatin. **(B)** Methotrexate. **(C)** Paclitaxel. **(D)** Doxorubicin. The differences were assessed by Wilcoxon tests.

The IC50 values of chemotherapy drugs were compared in order to provide treatment options for patients with OS. The IC50 values of cisplatin, methotrexate, and paclitaxel showed no significance between two risk groups (Figures 9A–C). Significantly, the IC50 value of doxorubicin was lower in high-risk group, suggesting that the patients in high-risk group may be more sensitive to doxorubicin (Figure 9D).

### 3.8. Functions of prognostic genes and their regulatory mechanisms

**FIGURE 10:**
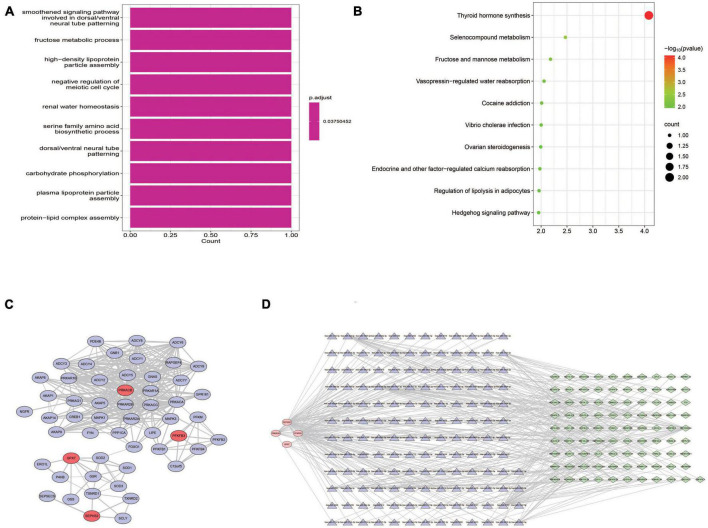
Functional analyses of prognostic genes. **(A)** Biological processes of GO functional analysis. **(B)** KEGG pathway analysis. **(C)** Protein-protein interaction network. **(D)** ceRNA regulatory network.

Both GO and KEGG analyses were performed to further identify the potential biological functions of these 4 prognostic genes. Several glycometabolism related biological processes such as fructose metabolic process, and carbohydrate phosphorylation were enriched (Figure 10A). KEGG analyses showed that these 4 genes were related to the pathway of thyroid hormone synthesis, fructose and mannose metabolism, and hedgehog signaling pathway (Figure 10B). In the PPI network, we found that these 4 prognostic genes could directly or indirectly interact with other 50 genes (Figure 10C). Furthermore, a lncRNA-miRNA-mRNA regulatory networks was conducted to identify the lncRNAs and miRNAs regulating the expression of prognostic genes. Finally, a ceRNA network composed by 4 glycometabolic genes, 148 miRNAs, and 91 lncRNAs, were identified (Figure 10D and Supplementary Table 6).

### 3.9. Validation the expression of prognostic genes in OS tissues

**FIGURE 11:**
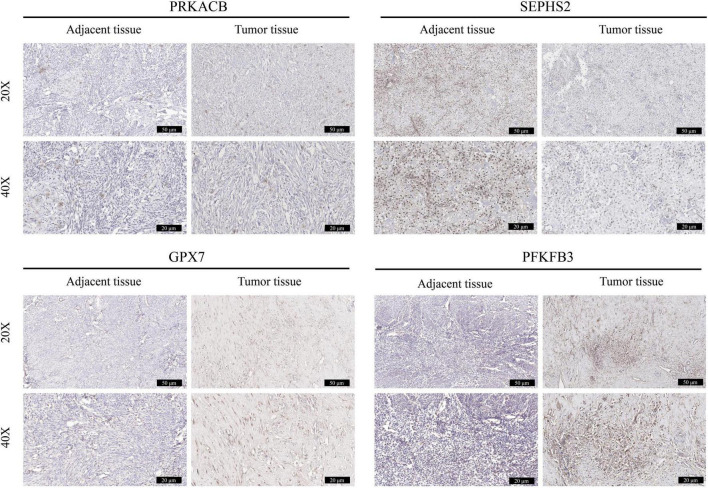
Validation the expression of prognostic genes in OS tissues. The immunohistochemical staining was performed to detect the expression of *PRKACB*, *SEPHS2*, *GPX7*, and *PFKFB3* between OS tissues and the corresponding adjacent tissues (magnification 20 and 40×).

The expression of these 4 prognostic genes were verified using immunohistochemistry (Figure 11). It was shown that *GPX7* and *PFKFB3* were up-regulated, and *SEPHS2* was down-regulated in OS tissues in comparison to those in adjacent normal tissues. However, the expression of *PRKACB* appeared to be no significant difference between OS tissue and adjacent normal tissue. These findings indicated that *GPX7*, *PFKFB3*, and *SEPHS2* may play critical roles in the development of OS.

## 4. Discussion

Glycometabolism in tumor cells is characterized by the enhanced glucose uptake and aerobic glycolysis (30). Aerobic glycolysis allows the conversion of glucose into pyruvate eventually contributing to the production of lactate. This energy metabolic reprogramming promotes energy generation and thus facilitates the proliferation, invasion, and chemoresistance of OS cells (31). Several factors, including glycolytic enzymes (e.g., GLUT1), oncogenes (e.g., KRT17), transcription factors (e.g., HIF1α), tumor suppressors (e.g., p53), and related signaling pathways, have been reported to regulate the glycometabolism of OS cells (32–34). Deep insight into these factors may help to formulate therapeutic strategies. In this study, a total of 4 glycometabolism related prognostic genes, including *PRKACB*, *SEPHS2*, *GPX7*, and *PFKFB3*, were identified using the univariate Cox regression analysis followed by a LASSO regression analysis. Then a glycometabolic gene signature was established based on these genes. Further analysis showed that the glycometabolic gene signature had a good performance and application value in predicting the prognosis of patients with OS. Meanwhile, this prognostic signature was significantly associated with immune microenvironment, and could provide options for the application of chemotherapy drugs. Functional enrichment showed that these 4 genes were enriched in multiple biological processes and pathways which have been demonstrated involving in the pathogenesis of OS. PPI and ceRNA network revealed that these 4 prognostic genes could interact with several genes, and their expression might be regulated by a variety of miRNAs and lncRNAs. These findings indicated that the novel glycometabolic gene signature may serve as a new diagnostic biomarker, and have the potential to predict the prognosis of patients with OS, which also may shed new light on the underlying mechanisms of OS from the perspective of glycometabolism.

Osteosarcoma (OS) is one of the highly aggressive tumors with high rates of metastasis and recurrence (1). The development of biomarkers for early diagnosis and predicting prognosis will facilitate the comprehensive treatment of patients with OS (35). Recently, several gene signatures have been established from different perspectives which could serve as diagnostic biomarkers and predict the prognosis of OS. These prognostic signatures can be divided into several categories, including immune related gene signatures (36, 37), hypoxic gene signatures (38, 39), cell death related gene signature (40–43), noncoding RNA related prognostic signatures (44, 45), epigenetics related gene signatures (46, 47), and metastasis associated gene signatures (48, 49). In addition, some metabolism related prognostic signatures have also been developed (50, 51). Glycometabolism, as one of the most important metabolic pathways in tumor, is of great importance to the initiation and progression of OS (10). Previous studies have showed that glycolysis related gene signatures are able to predict the prognosis of patients undergoing OS (52, 53). It is well known that the acquisition of energy in tumor cells also depends on oxidative phosphorylation (10). Hence, comprehensive analysis the roles of glycometabolism related genes will be more helpful to elucidate the correlation between glycometabolism and OS. In this study, we identified a novel prognostic gene signature based on 4 glycometabolic genes including *PRKACB*, *SEPHS2*, *GPX7*, and *PFKFB3*. Comprehensive analyses such as KM survival analysis, ROC curve, and nomogram indicated that this gene signature was robust and had a good performance in predicting the prognosis of patients with OS. These findings may enrich the study on the association between glycometabolic genes and the prognosis of OS.

In current study, bioinformatics analysis showed these 4 glycometabolic genes were differentially expressed between high- and low- risk groups. Significantly, the results of IHC staining also indicated the distinct difference of prognostic genes including *SEPHS2*, *GPX7*, and *PFKFB3* between tumor and adjacent normal tissues. Hence, we speculate these glycometabolic genes may play critical roles in the occurrence, and development of OS. However, due to the small sample size of OS tumor tissues included in this study, we failed to observe the significant difference of *PRKACB* between tumor and adjacent normal tissues. What is more, increasing studies have investigated their roles in the tumorigenesis. For instance, Lower *PRKACB* expression was found in tumor tissues and significantly associated with unfavorable overall survival in patients with colorectal carcinoma (54). It was shown that *SEPHS2* was elevated in breast tumor samples, and its overexpression was correlated with advanced tumor grade, suggesting that *SEPHS2* may serve as a prognostic marker and therapeutic target for patients with breast cancer (55). *GPX7* is a member of the glutathione peroxidase (GPx) family with weak GPx activity (56). *GPX7* has been confirmed to inhibit tumorigenesis, and may function as a tumor suppressor in multiple tumors (57, 58). *PFKFB3*, as a key regulator of glycolysis, has been implicated in tumorigenesis, angiogenesis chemoresistance, and tumor microenvironment (59). Furthermore, it was shown that upregulated *PFKFB3* could accelerate cell growth and metastasis, which may be a potential biomarker for OS (60, 61). Taking together, it is believed that the prognostic value of this signature may be attributed to the potential impacts of these glycometabolic genes to the tumorigenesis of OS.

The tumor immune microenvironment has long been shown to be strongly correlated with tumor development, recurrence and metastasis (62). Previous studies have suggested that Warburg effect participates in immunomodulation in the tumor microenvironment, and promotes immune evasion by against macrophage immunosurveillance (63, 64). However, little is known about the association between glycometabolism and immune microenvironment in OS. Herein, GSEA was performed and found that DEGs were enriched several immune related biological functions and pathways, such as in activation of innate immune response, adaptive immune response, antigen processing and presentation, natural killer (NK) cell mediated cytotoxicity, T cell receptor signaling pathway, and B cell receptor signaling pathway. ssGSEA analysis was conducted to further determine the correlations between glycometabolism and immune infiltration. The ssGSEA results indicated that a total of 26 immunocytes, including activated B cell, activated CD8 T cell, NK cell, and activated dendritic cell, were lower infiltration in high-risk OS samples than that in low-risk OS samples. Further analysis showed that significant links were found between these 4 glycometabolic genes and multiple immunocytes. Among these cells, NK cells, as a type of lymphocytes of the innate immune system, are able to recognize OS cells, release cytokines, and induce the cell lysis via multiple mechanisms (65). Enhancing NK cell-versus-OS effect has become a promising treatment for OS (66). Dendritic cells (DCs) are known as the central regulators of the adaptive immune response, and are essential for T cell mediated cancer immunity. It has been demonstrated that OS immunotherapy using a DCs-fused tumor vaccine could significantly facilitate T cells proliferation, and promote the tumor-cytotoxic activity of cytotoxic T cells (67). The combination of DCs and anti-glucocorticoid-induced tumor necrosis factor receptor antibodies had the ability to enhance systemic immune responses, and inhibit primary OS growth (68). In addition, we found that the decrease in immune score, stromal score, and ESTIMATE score while the increase in tumorpurity was detected in high-risk group. Stromal cells and immune cells are considered as the main components of tumor microenvironment, which is essential for tumorigenesis, invasion and immune infiltration (69). The increase of both stromal cells and immune cells in tumor environment facilitates immune resistance and immune escape, and the OS patients with higher stromal and immune scores may be appropriate for immune checkpoint inhibitor treatment (70, 71). Hence, these findings indicated that the patients with low- risk scores may obtain more benefit from immune checkpoint inhibitor treatment. Taking together, this glycometabolic gene signature may contribute to estimate the immune microenvironment of OS samples, and thus help formulate individualized anti-tumor immunotherapies.

It is known that 4 agents, including cisplatin, methotrexate, paclitaxel, and doxorubicin, are regarded as the first-line chemotherapy drug regarding OS treatment. Nevertheless, numerous studies have found that the resistance to these drugs can lead to unfavorable outcomes of patients with OS (72). Currently, the prediction of the responses to chemotherapies is still limited by lack of effective biomarkers. In present study, we investigated the drug sensitivity between two risk groups, and the results showed that the patients in high-risk group were more sensitive to doxorubicin. As mentioned above, an increase of tumorpurity score was found in high-risk group, indicating that there were much more OS tumor cells in high-risk samples when compared to that in low-risk samples. Meanwhile, the correlation between risk scores and metastasis suggested that higher proportion of patients with metastasis was found in high-risk group in comparation to that in low-risk group. Hence, we speculate that the patients with high tumorpurity score or metastatic OS may be more effective to the chemotherapy with doxorubicin. These findings indicated that the risk score model may help guide the selection of chemotherapy drugs. Although multiple studies have demonstrated the pharmacological effect on OS treatment, it is necessary to conduct large sample size of cohort study to explore the clinical effectiveness of doxorubicin for the patients with high-risk scores.

What is more, we also tried to identify more details about these 4 prognostic genes in the pathogenesis of OS. GO functional and KEGG analyses showed that these 4 prognostic genes were enriched in the biological processes of fructose metabolic process, carbohydrate phosphorylation, and the pathways of thyroid hormone synthesis, fructose and mannose metabolism, and hedgehog signaling pathway. These biological processes and pathways may participate in the regulation of glycometabolism in OS. The PPI network suggested that these 4 prognostic genes were able to directly or indirectly interact with other 50 genes. Some of these genes have been shown involving the initiation and progression of OS. For instance, Wang and Sun (73) demonstrated that inhibition of FOXO1 was able to suppress both proliferation and metastases in OS cells, indicating that FOXO1 may be a potential target for the treatment of OS. In addition, CREB1 could lead to OS progression and metastasis through promoting epithelial-mesenchymal transition (74). Hence, deeper insight into these genes in the network may help uncover the underlying mechanisms of these 4 prognostic genes in the development of OS. CeRNA network is regarded to be a prevalent form of post-transcriptional regulation of gene expression in mammals (75). Message RNA (mRNA), long non-coding RNA (lncRNA), pseudogene, and circular RNA (circRNA) can affect the stability or translation activity of target RNAs by competitive combination with microRNA (miRNA). Increasing studies have confirmed that ceRNA regulatory network is widely involved in the occurrence and development of OS (76). To further understand the upstream mechanisms of prognostic genes, a ceRNA network was performed to identify the target miRNAs and the corresponding lncRNAs. The results showed that a total of 148 miRNAs and 91 lncRNAs were linked with these four prognostic genes. These findings may facilitate a better understanding of these 4 prognostic genes in the pathogenesis of OS.

Some limitations of this study should be interpreted. First, both training cohort and testing cohorts used in current study contain relatively small sample size. Meanwhile, some clinical features including tumor size and grade of OS patients are missing in TARGET dataset. Hence, we did not perform stratification analysis to investigate the correlations between risk score and tumor size and grade. Second, due to limited clinical tumor samples used in current study, we failed to find distinct difference of *PRKACB* between tumor and adjacent normal tissues. Given that low expression of *PRKACB* has been demonstrated leading to unfavorable clinical outcomes in patients undergoing breast cancer and colorectal carcinoma (54, 77), we speculate that the low expression of *PRKACB* may contribute to the occurrence and development of OS, thereby bringing about inferior clinical outcomes. However, no studies have reported the functions of these prognostic genes including *PRKACB*, *SEPHS2*, and *GPX7* in the pathogenesis of OS. Therefore, more OS tumor samples and multiple OS cell lines should be applied to determine the expression of these prognostic genes and their roles in the tumorigenesis of OS.

In summary, our research identified a novel signature based on four glycometabolic genes, and constructed a risk score model that can predict the survival, immune infiltration, and chemosensitivity of patients with OS. These findings may provide new insights into the role of glycometabolic genes in the molecular mechanisms of OS, and help develop novel diagnostic and therapeutic strategies.

## Data availability statement

The original contributions presented in this study are included in the article/Supplementary material, further inquiries can be directed to the corresponding authors (78).

## Ethics statement

The studies involving human participants were reviewed and approved by the Ethics Committee of Affiliated Hospital of Guizhou Medical University (No. GZYD003-201753035). The patients/participants provided their written informed consent to participate in this study.

## Author contributions

HS and KL conceived the study. FW and KY performed the bioinformatic and statistical analysis. RP participated in collecting the data from online database. ZX and PL participated in figures and charts drawing. YX supervised the implement of the current study. HS, FW, and KL provided fund and prepared the original draft. HS reviewed and edited the manuscript. All authors contributed to the article and approved the submitted version.

## Footnotes

1
https://ocg.cancer.gov/programs/target

2
https://www.ncbi.nlm.nih.gov/

3
http://www.broad.mit.edu/gsea/msigdb

## Supplementary material

Supplementary Figure 1 Non-metastasis and metastasis cases between low- and high-risk groups in TARGET dataset.
