# Supplementary figures and images for: A glycometabolic gene signature associating with immune infiltration and chemosensitivity and predicting the prognosis of patients with osteosarcoma
Source: Front Med (Lausanne). 2023 May 24;10:1115759. doi: 10.3389/fmed.2023.1115759 (PMC10244582; doi:10.3389/fmed.2023.1115759)

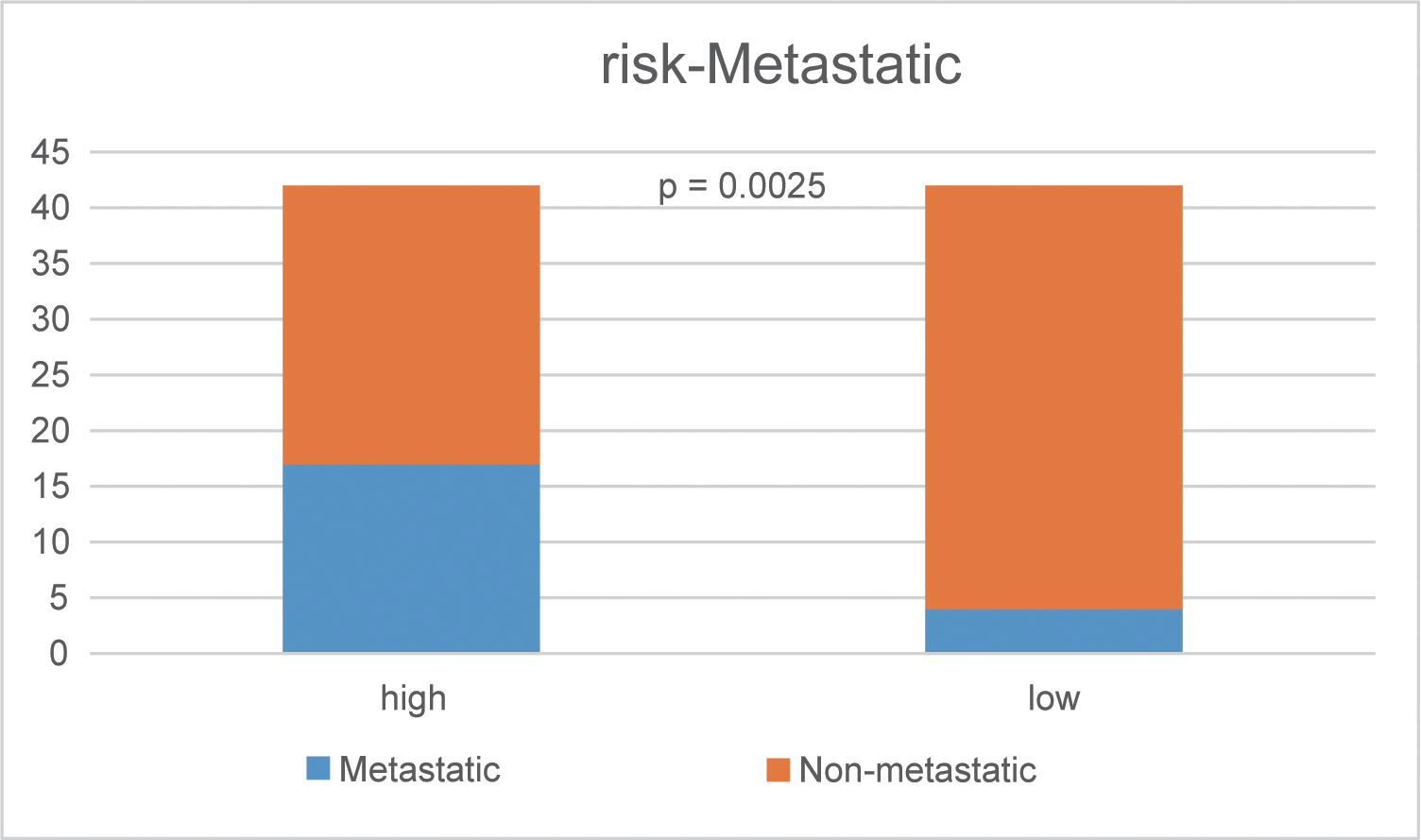

Supplement: Supplementary Figure 1 — Non-metastasis and metastasis cases between low- and high-risk groups in TARGET dataset. [file Image_1.TIF]
